# Real-time emotional dynamics and public discourse on Sina Weibo following the first imported mpox case in mainland China: A sentiment analysis study

**DOI:** 10.1371/journal.pone.0354150

**Published:** 2026-08-07

**Authors:** Zhanyan Li, Yi Li, Ke Li, Zhuoxiao Li, Chunbei Zhou, Hong Xiao

**Affiliations:** 1 Chongqing Medical University-University of Leicester Joint Institute, Chongqing Medical University, Chongqing, China; 2 Chongqing Center for Disease Control and Prevention, Chongqing, China; 3 Academy of Chip Technology, China electronic technology group corporation, Chongqing, China; 4 School of Public Health, Southeast University, Nanjing, China; 5 Department of Disease Prevention and Control, Daping Hospital, State Key Laboratory of Trauma and Chemical Poisoning, Hospital Infection Monitoring and Control Key Laboratory of Chongqing Education Commission of China, Army Medical University (Third Military Medical University), Chongqing, China; 6 College of Public Health, Chongqing Medical University, Chongqing, China; Federal University Otuoke, NIGERIA

## Abstract

**Background:**

The human mpox has become a significant public health threat; however, little is known about real-time public emotional responses to mpox in China.

**Methods:**

We collected comments from the top 40 popular posts containing the keyword “Chongqing mpox” on Sina Weibo within 48 hours following the announcement of the first imported mpox case in mainland China (September 16, 2022). Using natural language processing (NLP), we applied the BosonNLP sentiment dictionary to assign emotional values (EVs) to each comment. Latent Dirichlet Allocation model was used to identify key public concerns, and Spearman correlation analysis was conducted to examine relationships between engagement metrics (comments, likes, responses) and EVs.

**Results:**

The 40 popular posts received 5,107 comments, 144,940 likes, and 7,358 responses, respectively. Weibo users’ comments reached a peak within two hours after the announcement (hourly data) and decreased markedly after four hours. Users with the most comments came from Guangdong, Shanghai, Beijing, Zhejiang, and Chongqing. The public was most concerned about attitudes and the transmission mode of the mpox. Sentiment analysis showed that the comments were mostly negative (76.13%). The EVs of the negative comments were 32.31 times higher than those of the positive comments. The numbers of comments, likes, and responses were all significantly associated with EVs (p < 0.001).

**Conclusions:**

Public sentiment following the first imported mpox case in mainland China was predominantly negative and highly concentrated within the first four hours. Findings underscore the need for timely, region-sensitive risk communication during emerging outbreaks.

## 1. Introduction

Mpox is a zoonotic disease caused by the mpox virus, which belongs to the poxvirus family [1,2]. The virus can be transmitted directly through contact with lesions, respiratory secretions, or prolonged close person-to-person contact [3]. The first human case of mpox was detected in the Democratic Republic of Congo in 1970 [4]. Since then, human mpox has been considered endemic in Central and West Africa, with regular outbreaks in poorer and neglected communities [5,6]. Sporadic outbreaks caused by mpox did not attract attention until May 2022, when a cluster of mpox cases involving dozens of UK residents was reported [7]. On 23 July 2022, the World Health Organization declared the mpox outbreak a public health emergency of international concern [8]. This is the first time human mpox has occurred on a large scale way across continents, with sustained local person-to-person transmission [9]. It is worth noting that high prevalences of HIV and other sexually transmitted infections have been reported in the global mpox outbreak, which has disproportionately affected primarily gay, bisexual, and other men who have sex with men (MSM) [10].

The first imported mpox case in mainland China was reported in Chongqing on September 16, 2022 [11]. This incident immediately sparked intense discussion on Chinese social media. A cross-sectional online survey found that the Chinese public had insufficient mpox knowledge [12], while another survey of MSM in China showed that 69.9% reported awareness of mpox [13]. Given that this incident triggered widespread emotional expression on social media, real-time monitoring and analysis of public sentiment are particularly important. Public emotions play a central role in the dissemination of information during public health emergencies. According to the Crisis and Emergency Risk Communication model, emotional responses, such as fear, anxiety, and anger, directly influence public risk perception, information-seeking behavior, and adoption of protective measures [14]. The rapid spread of negative emotions can lead to panic, stigmatization, and even irrational behaviors, thereby hindering effective epidemic control efforts. Therefore, real-time monitoring and guidance of public emotions during the critical window following an outbreak announcement is a core task of risk communication. However, existing research has largely focused on the content and themes of public discourse, with insufficient attention paid to emotional dynamics themselves, particularly in non-Western social media environments.

Sina Weibo, as China’s leading real-time social media platform with over 462 million users, provides a unique data source for studying public emotional responses [15]. Weibo comments not only reflect the public opinion on events but also contain rich emotional information, which can be classified as positive, negative, or neutral [16,17]. Emotional transmission on the Internet often directly affects behavior change. Sentiment analysis, using natural language processing (NLP) and text mining to analyze emotionally subjective texts [18], has been widely applied in public health research. One of the important applications is to help guide public opinion and calm emotions during major events (such as extreme events, disaster events, etc.) [19]. While an increasing number of studies have examined public sentiment during the COVID-19 pandemic [20], similar research in the context of mpox outbreaks remains limited, particularly in the Chinese social media environment. A preliminary analysis of this event was published in Chinese by our group using the Weibo platform’s built‑in sentiment module [21]. The present study differs from that prior work in terms of both methodology and dataset. Methodologically, we used independent data collection, BosonNLP‑based scoring with manual validation, temporal segmentation, Latent Dirichlet Allocation (LDA) topic modeling, and correlation analysis. Regarding the dataset, we analyzed originally crawled comment texts rather than aggregated platform data. The report of the first imported mpox case in mainland China provides a valuable opportunity to investigate real-time emotional dynamics in non-Western social media environments.

This study analyzed comments on popular Sina Weibo posts using NLP and sentiment analysis techniques to address the following three research questions: (1) How does emotional valence change over time? (2) What topics dominate discourse? (3) How does engagement correlate with sentiment? By addressing these questions, this study aims to provide empirical evidence for government and health authorities to formulate targeted public opinion guidance, emotional reassurance, and risk communication strategies in the early stages of public health emergencies. The findings can contribute to the growing field of digital public health surveillance and inform the development of sentiment-aware communication protocols for emerging infectious diseases.

## 2. Methods

### 2.1. Data source

#### 2.1.1. Search strategy and data collection.

**Fig 1:**
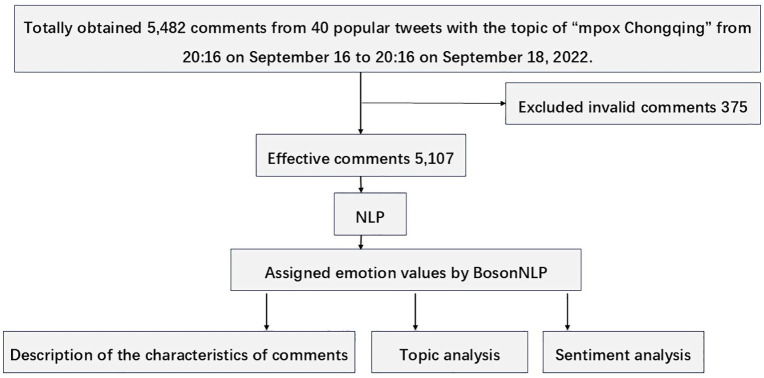
Study design.

The data of this study were obtained from the Sina Weibo (https://weibo.com). The observation period started from the release of the news “An imported case of mpox from abroad was reported in Chongqing, China” by “China News Service” at 20:16 on September 16, 2022, and ended 48 hours later (20:16 on September 18, 2022). Using a custom Python web crawler (V3.10.7), we searched for all original posts on topics containing the exact keyword “重庆 猴痘” (Chinese for “Chongqing mpox”) published during this 48-hour window. The study design is shown in Fig 1.

#### 2.1.2. Inclusion and exclusion criteria.

Posts were included if they were: (1) original posts (not reposts); (2) written in Chinese; and (3) directly related to the mpox event. Posts that were advertisements, spam, or contained only images/videos without text were excluded. From the initial pool of posts meeting these criteria, we ranked them by total engagement, defined as the sum of the number of comments (NC), likes (NL), and responses (NR). We selected the top 40 posts with the highest engagement for further analysis. The decision to focus on the top 40 posts was based on the observed engagement distribution. Posts ranked 41st and below each received fewer than 5 comments, providing insufficient textual data for meaningful sentiment analysis. Collectively, the top 40 posts accounted for over 95% of the total public engagement on this topic during the 48-hour observation period. A flow diagram illustrating the complete data selection process is provided in S1 Fig.

#### 2.1.3. Comment extraction and preprocessing.

From these 40 posts, we extracted all direct comments (excluding secondary replies to comments) published within the 48-hour window. A total of 5,482 comments were collected. The raw comment data were then preprocessed to remove noise that does not reflect user’s emotional inclinations, including images, emojis, URLs, forwarding text, user mentions, duplicate comments, and comment consisting solely of punctuation or meaningless characters. After preprocessing, 5,107 comments remained and were defined as valid comments for sentiment analysis (effective rate: 93.16%). The retained data included comment release time, user’s IP-based geographic region, cleaned comment text, NL, and NR.

#### 2.1.4. Geographic classification.

Regions were categorized based on users’ IP addresses displayed on Weibo, covering all provinces and cities in China (including Hong Kong, Macao, and Taiwan). Comments from overseas or from users with hidden IP addresses (labeled as “others”) by the platform were excluded from geographic analysis due to their minimal proportion (<1% of total comments).

#### 2.1.5. Compliance with source terms and conditions.

The data collection and analysis methods strictly adhered to Sina Weibo’s Terms of Service and Robot Exclusion Protocol. Only publicly accessible posts and comments were collected; no private user information was accessed. All data were anonymized prior to analysis to protect user privacy, and the study process conforms to the ethical guidelines for internet-based research.

### 2.2. Text sentiment

#### 2.2.1. Text preprocessing.

The R (V4.3.3) was applied for NLP of the review comments. Preprocessing steps included: (1) removing the meaningless characters and stop words (e.g., “a”, “the”) using standard stops word lists [22]; (2) tokenizing the remaining text into meaningful keywords using the jiebaR package of R [23]. For example, the sentence “Go China!” was segmented into “Go”, “China”, and “!”.

#### 2.2.2. Sentiment dictionary.

Sentiment analysis was performed using the BosonNLP sentiment dictionary, a widely used lexicon for Chinese text sentiment analysis [24]. The dictionary was developed by Boosen Technology, a Chinese NLP company, and is constructed specifically for social media content, including Weibo, news comments, and forums. It contains over 170,000 entries, each annotated with part-of-speech and sentiment polarity (positive, negative, or neutral). The absolute value of the scores reflects the intensity of sentiment. The dictionary is created through integration, deduplication, conversion, and supplementation of multiple lexical resources, with manual verification to ensure accuracy [25]. Its coverage of internet slang, neologisms, and colloquial expressions makes it particularly suitable for analyzing Weibo comments.

#### 2.2.3. Calculation of emotional values (EVs).

The emotional value of each comment was calculated using a rule-based scoring algorithm that considers emotional words, negation words, and degree adverbs. The algorithm follows these principles: (1) Each emotional word in the dictionary has a pre-assigned sentiment scores, ranging from −6.7 to 6.4. Positive words have scores >0, negative words have scores <0. Some examples of the positive and negative words are listed in S1 Table. (2) When a negation word (e.g., “不” [not], “没有” [no]) precedes an emotional word, the polarity is reversed, and the score is multiplied by −1. For multiple consecutive negations, odd counts reverse polarity while even counts restore original polarity. A negation word is considered to modify an emotional word only if it appears within a sliding window of up to four words before the emotional word (excluding stop words). (3) Degree adverbs (e.g., “非常” [very], “极其” [extremely], “有点” [slightly]) modify the intensity of “非常” emotional words. Each degree adverb has an intensity weight α(d) (e.g., “非常” = 1.8, “有点” = 0.5) (S2 Table). The emotional word score is multiplied by this weight. For a comment containing emotional words after negation and weighting adjustments [26], the total EV is calculated as:

EV =Σ[α(dj)×(−1)^(Nj)× s(wj)] for j=1 to n

α(dⱼ) is the intensity weight of the degree adverb modifying wⱼ;

Nⱼ is the number of negation words preceding wⱼ;

s(wⱼ) is the base score of emotional word wⱼ.

For example, the comment (original Chinese) is “这种病毒非常可怕.” The calculation follows the standard procedure: (1) emotional word identification from BosonNLP dictionary (base score −2.1 for “可怕”); (2) degree adverb weighting (α = 1.8 for “非常”); (3) negation checking within the four-word window (none present); (4) total EV = −3.78. The absolute value of EV reflects emotional intensity; higher absolute values indicate stronger emotional expression. A comment EV of 0 indicates neutral emotions, less than 0 indicates negative emotions, and greater than 0 indicates positive emotions.

#### 2.2.4. Validation check.

To validate the reliability of the BosonNLP-based sentiment analysis, we conducted a manual validation on a random sample of 500 comments (approximately 10% of the dataset). Two independent researchers with expertise in Chinese linguistics and sentiment analysis manually coded each comment as positive, negative, or neutral, following a standardized coding protocol. The researchers were blinded to the BosonNLP-derived scores during coding. Inter-rater reliability between the two human coders was assessed using Cohen’s κ. The obtained κ value was 0.78 (95% confidence [CI] interval: 0.74–0.82), indicating substantial agreement according to Landis and Koch’s benchmarks. This high level of agreement confirms the consistency and reliability of the manual coding scheme. Then, we compared the manual coding consensus (agreed-upon sentiment from both coders) with the BosonNLP-derived sentiment classification. The agreement between human consensus and BosonNLP was also substantial, with Cohen’s κ = 0.72 (95% CI: 0.68–0.76). This validation demonstrates that the BosonNLP dictionary performs reliably on our specific dataset of mpox-related Weibo comments.

To specifically assess the tool’s performance on discourse features relevant to the mpox context, we stratified the same 500-comment validation sample by content type: (1) standard Chinese expressions (n = 261); (2) comments containing internet slang or neologisms (n = 124); and (3) comments focused on transmission routes or stigmatizing language related to mpox (n = 82). The remaining comments (n = 33) with mixed or unclear categories were excluded from stratified analysis. The agreement between manual coding and BosonNLP remained substantial across all three strata: κ = 0.79 for standard Chinese, κ = 0.68 for internet slang/neologisms, and κ = 0.71 for transmission-related discourse. While performance was slightly lower for non-standard expressions, the overall consistency confirms that BosonNLP is acceptable for the primary analyses in this dataset.

### 2.3. Data analysis

#### 2.3.1. Descriptive and statistical analysis.

Excel 2019 was used to input data, and R (V4.3.3) was employed to examine the descriptive characteristics of comments. The Kolmogorov-Smirnov test was used to assess the normality of the data. Since the EVs did not follow a normal distribution, non-parametric tests were applied for subsequent analyses.

#### 2.3.2. Topic modeling.

To identify the main themes of public discourse in Weibo comments, we used LDA, a generative probabilistic model for uncovering latent topics in text corpora [27]. LDA with Gibbs sampling was implemented using the “topicmodels” package in the R (V.4.3.3). The Gibbs sampling algorithm was run for 2,000 iterations with a burn‑in period of 1,000 iterations. The log‑likelihood was monitored every 200 iterations, and convergence was considered reached when the change in log‑likelihood between successive checkpoints was less than 1.0. Determining the appropriate number of topics (k value) is a critical step in LDA modeling. We systematically evaluated models with k values ranging from 2 to 15, incrementing by 1. For each k, we calculated two standard model fit metrics. The perplexity and coherence scores for each k value were calculated and plotted (S2 Fig and S3 Table). Lower perplexity indicates better predictive performance and higher coherence scores indicate more coherent and interpretable topics. Based on the comprehensive evaluation of two indicators, the k = 5 was selected as optimal model. The model output provided for each topic the top words ranked by their probability of belonging to that topic. The labeling of topics was conducted inductively by two independent researchers, both with expertise in public health and social media analysis. Each researcher independently reviewed the top 20 most representative words (highest probability) for each of the five topics. Based on the word lists, each researcher proposed a descriptive label for each topic that captured the underlying theme. The two researchers met to compare their proposed labels. Disagreements were discussed and resolved through consensus. For any unresolved disagreements, a third researcher served as an arbitrator. The final set of topic labels was reviewed by all authors to ensure they accurately reflected the content of the comments. To assess topic stability, we re‑ran the LDA model with k = 5 using 10 different random seeds. For each run, we computed the average pairwise topic similarity using the Jaccard coefficient of top‑10 words per topic.

#### 2.3.3. Temporal and spatial analysis of emotions.

Comments were aggregated by hour to examine temporal patterns of engagement and emotional expression. To describe the temporal and spatial characteristics of emotions, we divided the 48 hours after the event into three periods based on observed comment volume patterns [28]. Stage 1 encompassed the initial 0–4 hours following the report (peak engagement period); Stage 2 covered the subsequent 4–12 hours (rapid decline period); Stage 3 extended from 12–48 hours post-report (plateau and gradual decay period). Because the periods were not equally spaced, we defined the EV per hour (EV/h) to reflect the intensity of emotional expression over time. EV/h was calculated as the cumulative EV within a fixed period (sum of EVs of all comments in that period) divided by the duration of the period (4 hours for Stage1, 8 hours for Stage 2, and 36 hours for Stage 3). The Kruskal–Wallis test was used to compare the EV/h across the three periods, followed by Dunn’s post-hoc test with Bonferroni correction for pairwise comparisons. Effect sizes were calculated to quantify the magnitude of differences between periods. The BioLadder platform was used to visualize the spatiotemporal distribution of emotions on a map of China.

#### 2.3.4. Correlation analysis.

All correlations were computed at the post level (n = 40 popular posts). For each post, we aggregated the NC, NL, and NR, as well as the EV of all direct comments on that post (including separate sums for positive and negative EVs). Spearman correlation was then applied to assess the relationships between these post-level metrics, drawn with Origin 2024. To control for the increased risk of type I error due to multiple comparisons, we applied the Bonferroni correction. With 9 correlation tests conducted in the main analysis (3 engagement metrics × 3 EV categories), the adjusted significance threshold was set at p < 0.0056 (0.05/9). Correlations reported as significant met this adjusted threshold.

All statistical tests were two-sided, and p < 0.05 was considered statistically significant.

### 2.4. Ethical approval

This study was a non-interventional research based on publicly available online texts from Sina Weibo. No questionnaires, interviews, or interactions with participants were employed, and no sensitive personal information was collected. This research was conducted in accordance with the Association of Internet Researchers Ethical Guidelines 3.0 [29] and the British Psychological Society Ethics Guidelines for Internet-mediated Research [30]. Following these guidelines, formal ethics committee approval was not required. Furthermore, the following measures were implemented to ensure confidentiality: (1) All personally identifiable information (usernames, IP addresses) was removed; geographic analysis was conducted only at the province level. (2) No verbatim comments are included in this manuscript to prevent backwards searching that could compromise anonymity. (3) The anonymized dataset is stored on password-protected servers and shared publicly via Figshare with no identifying information (see Data Availability statement). Data collection complied with Sina Weibo’s Terms of Service.

## 3. Results

### 3.1. Characteristics of the comments

**Fig 2:**
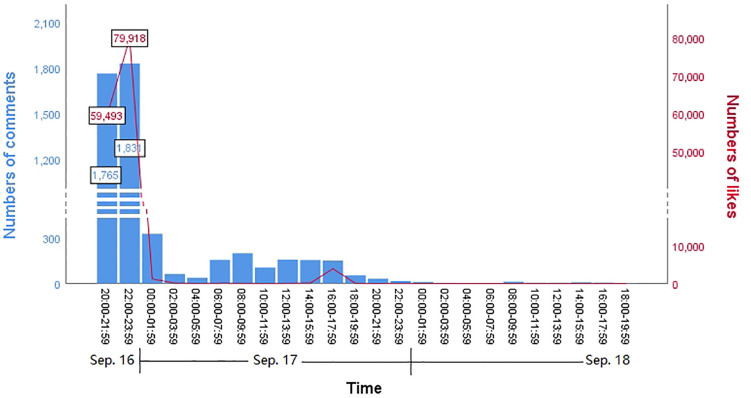
Temporal distribution of comments and likes following the event announcement. Data are aggregated by hour. The x-axis shows hours after the news release (20:16, September 16, 2022). The left y-axis represents the number of comments (blue line), and the right y-axis represents the number of likes (red line).

**Fig 3:**
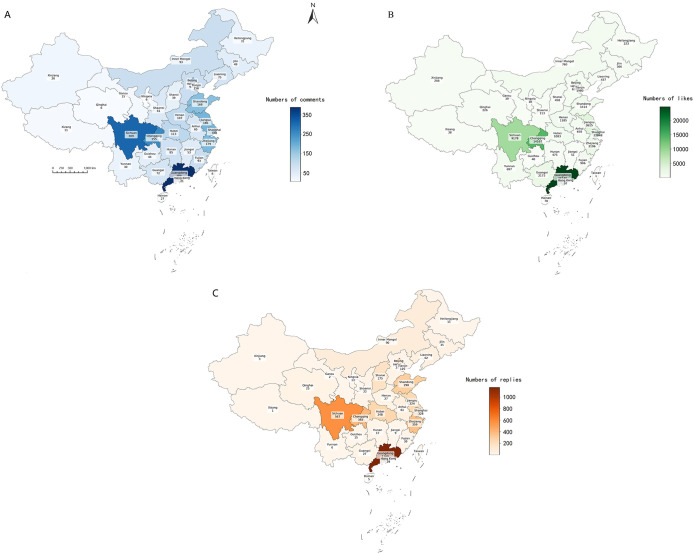
Geographical distribution of comments, likes, and responses in China. Color intensity represents the total number of valid interactions from each province. (A) Numbers of comments; (B) Numbers of likes; (C) Numbers of responses. Base map: Natural Earth (public domain).

The 5,107 valid comments received a total of 144,940 likes and 7,358 responses, with the most commented receiving 19,685 likes and 702 responses. As shown in Fig 2, the NC, NL, and NR peaked in the first four hours (Stage 1) and then decreased significantly. The NC and NL in the first four hours accounted for 72.27% and 96.68% of the total, respectively. Fig 3 shows the geographical distribution of comments. The regions with the most cumulative NC, NL, and NR were Guangdong, Shanghai, Beijing, Zhejiang, Chongqing, and Sichuan.

**Fig 4:**
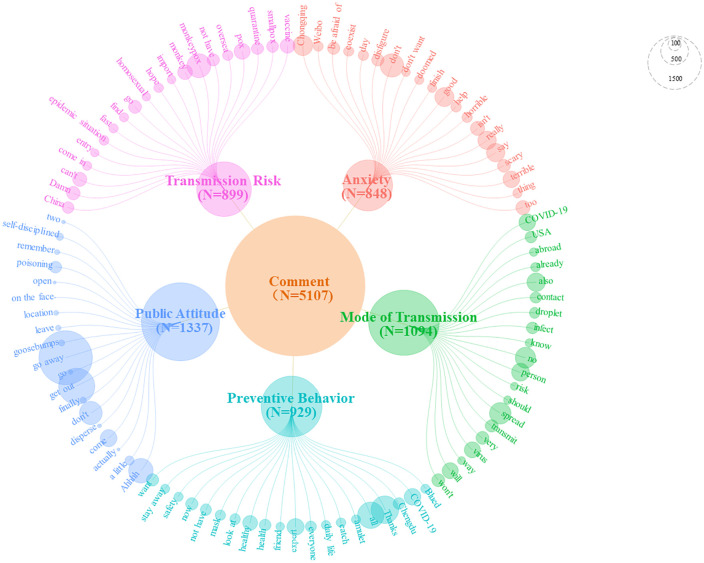
Visualization of LDA topic clustering. Each topic is associated with representative words in the outermost layer. Circle size denotes word frequency with each topic.

The results of the clustered words generated by the topic analysis represented the Weibo users’ perspectives on the event, as shown in Fig 4. The main clustered words from the comments were “Public Attitude” (26.18%) and “Mode of Transmission” (21.42%). Under the word “Public Attitude”, “go away” and “get out” were the most frequent words. Under the word “Mode of Transmission”, “COVID-19” and “spread” were the most frequent words. From the document-topic probability matrix, the mean proportion (Standard Deviation, SD) of each topic across the entire comment set was: Public Attitude 0.31 (0.12), Mode of Transmission 0.27 (0.10), Prevention Behavior 0.18 (0.09), Transmission Risk 0.13 (0.08), and Anxiety 0.11 (0.07). The mean pairwise Jaccard similarity of the top‑10 words per topic across runs was 0.89 (SD = 0.04), indicating high stability.

### 3.2. Results of sentiment analysis

**Fig 5:**
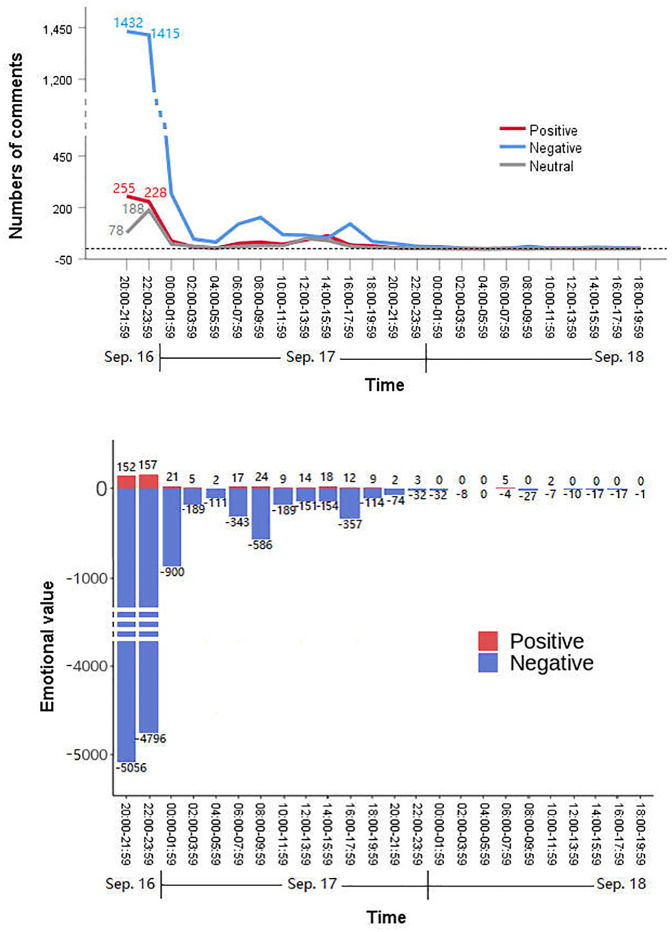
Time distribution of comment volume and emotional value. The lines represent the number of comments per hour, and the bars represent the emotional values per hour. Vertical lines divide the 48-hour period into three stages.

All valid comments were assigned EVs, and the values did not follow a normal distribution (D = 0.173, p < 0.001). The total EV of all comments (accumulate the sentiment score of each valid comment) was −12,724, with one of the most negative comment (the absolute value was the highest) scoring −113.30. The distribution of EVs revealed two key patterns. First, negative comments dominated both in quantity (76.13%) and cumulative intensity (total EV = −13,174.41), with negative EVs exceeding positive EVs by a factor of 32. Second, negative comments were expressed more intensely than positive comments (median EV: −2.31 vs. 0.38). The most negative comment (EV = −113.30) was approximately 15 times more intense than the average negative comment, reflecting extreme emotional expression by some users (Table 1). The temporal distribution of comments’ EVs is shown in Fig 5. Both positive and negative emotions exhibited a relatively slow decline during the first two hours, with a more noticeable drop occurring after that period. Within four hours of the event, the numbers and values of negative comments were much higher than positive comments. Negative comments scored 32.31 times higher than positive comments.

**Table 1:** Characteristic distribution of emotional value in comments.

	N (%)	Total EV	Mean EV (SD)	Median EV (IQR)	Min	Max	Practical interpretation
**All comments**	5,107 (100.00)	−12724.13	−2.49 (3.97)	−1.88 (−3.43 to −0.08)	−113.30	7.55	Overall sentiment was strongly negative
**Positive (>0)**	760 (14.88)	450.28	0.59 (0.73)	0.38 (0.17 to 0.72)	0.01	7.55	Positive expressions were mild in intensity
**Neutral (=0)**	459 (8.99)	0	0	0	0	0	No emotional expression detected
**Negative (<0)**	3,888 (76.13)	−13174.41	−3.39 (4.15)	−2.31 (−4.18 to −1.41)	−113.30	0	Negative comments were both more numerous and more intensely expressed

**Fig 6:**
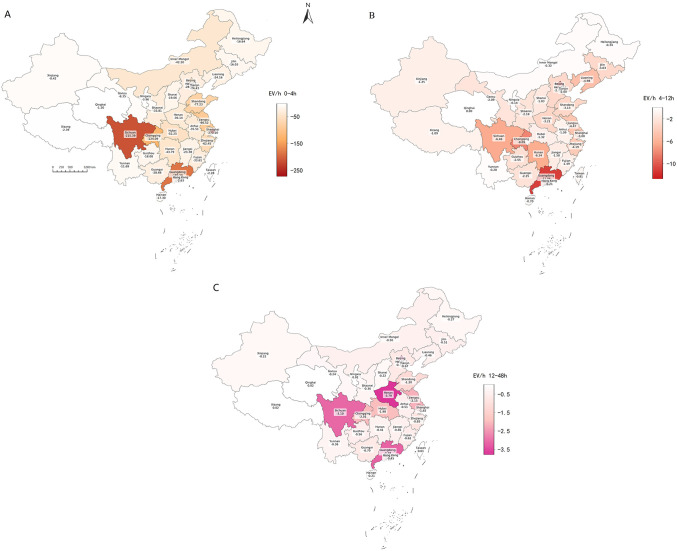
Spatial and temporal distribution of EV/h across China. Color gradient represents the EV/h for each province. The darker the color is, the more negative the emotion is. Base map: Natural Earth (public domain).

In terms of EVs of different areas in China, the top negative areas were Shanghai (−1175), Sichuan (−1025), Guangdong (−945), Beijing (−660), and Chongqing (−634), accounting for 50.22% of the total value. In terms of time segments, all regions of China in Stage 1 showed negative emotions, which weakened in Stages 2 and 3. Among them, the EV/h in Shanghai decreased from 273.1 in Stage 1 to 3.0 in Stage 3. In the last stage, EV/h in Ningxia, Tibet, Qinghai, and Taiwan was greater than 0, indicating positive emotions (Fig 6). The EV/h differences among the three stages were statistically significant (H = 62.26, df = 2, p < 0.001), with a large effect size (ε² = 0.51).

### 3.3. Results of correlation analysis

**Fig 7:**
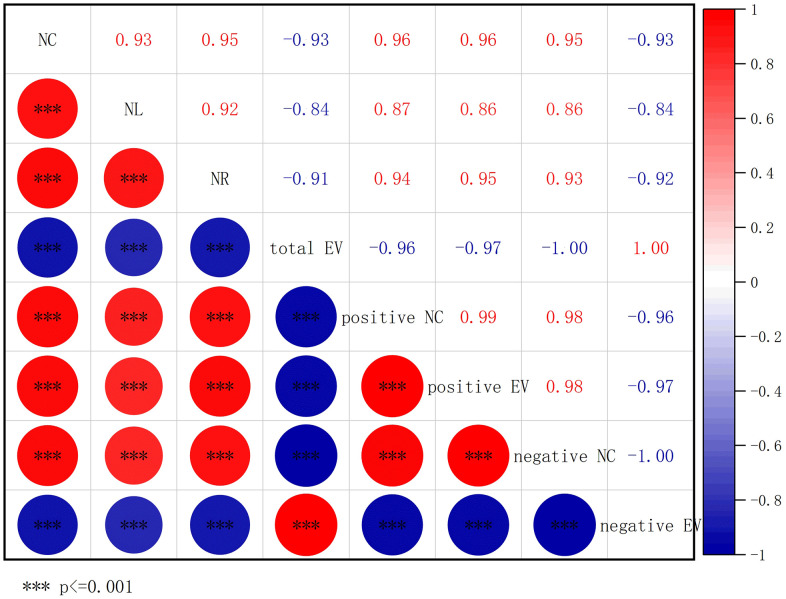
Correlation analysis between comments and emotional values. Values are Spearman’s rank correlation coefficients (ρ). NC: number of comments; NL: number of likes; NR: number of responses; EV: emotional value. Positive EV: total EV of positive comments; Negative EV: total EV of negative comments. Red represents positive correlation, blue represents negative correlation. Color intensity reflects correlation strength. * indicate correlations significant at the Bonferroni-corrected threshold of p < 0.0056.

Correlation analysis results showed that, at the post level (n = 40), the NC, NL, and NR were significantly associated with EVs, including positive emotion and negative emotion (Fig 7). The NC showed the strongest negative correlation with total EV (ρ = −0.93, p < 0.001), indicating that higher discussion volumes were associated with more negative overall sentiment. All reported correlations remained significant after Bonferroni correction (adjusted p < 0.0056).

## 4. Discussion

To our knowledge, this was the first study to evaluate the public’s emotional responses and evolving characteristics following a mpox hotspot event based on the original Weibo data. In our study, we observed that the volume of comments peaked within the initial four-hour period after the event was reported, and then decreased significantly. The participants in the discussion using Weibo exhibited a distinct geographical distribution, and the content of the comments focused on the public’s attitude and the transmission mode of mpox. The result of sentiment analysis indicated that an overwhelming majority of the comments expressed negative sentiments, which exhibited a significant correlation with the valid NC, NL, and NR. Our findings highlight the viability of utilizing Weibo hot posts and comments to gauge public emotions and suggest the importance of early emotional guidance and intervention after reporting a public health emergency.

Social medias, such as Weibo and WeChat, play a vital role in disseminating government information and guiding behavior during public health emergencies [31]. A study using popular Weibo text related to COVID-19 found that different stages of the epidemic correspond to different emotions [32]. Our previous analysis based on the micro-hotspots of the Weibo sub-section showed that the mpox epidemic triggered higher levels of neutral and angry emotions, and women’s emotions were more sensitive [21]. Our current research expanded the spatial-temporal variation characteristics of emotions and conducted an in-depth mining of the text of the comments.

In this study, the intense public discourse, including the NC, NL, and NR, decreased substantially within 4 hours of the peak. It lasted for a plateau period of 12 hours and then decayed slowly. This 4-hour peak pattern is consistent with observations during the COVID-19 pandemic on Chinese social media [28], suggesting it may reflect platform-specific attention dynamics and the rapid news cycle characteristic of Weibo, rather than a pattern unique to mpox. This rapid “attention spike” within four hours, followed by sharp decline, underscoring the critical window for initial risk communication. The difference is that the mpox news report involved one imported case, which triggered less widespread online discussion than the novel coronavirus outbreak, and the discussion lasted significantly shorter. This may be related to the fact that the news was released at night, close to the rest time of the public. Additionally, mpox is not a new infectious disease; its transmission modes are well-documented, and the pathogenesis is well-understood [33,34], leading to potentially lower levels of public concern. The analysis showed that emotional responses peaked within the first 4 hours after the news was released. It would be valuable to suggest that official communication and emotional reassurance efforts should be intensified during that window. The government must make accurate decisions and adopt proper preventive measures, the hospitals and public health agencies should prepare for medical treatment, epidemiological investigation, and popularization of science, and the public should know how to protect themselves efficiently [35,36].

Our study also found that the geographical distribution of users who participated in discussions around the event on Weibo showed unexpected results. In addition to Chongqing, the city of the incident, and its neighboring Sichuan province, Weibo users from Guangdong, Shanghai, and Beijing also exhibited high levels of engagement and emotional expression. This concentration in raw engagement metrics in economically developed regions is consistent with higher internet penetration rates in these areas (>85% in these areas vs. national average 73%) [37], but may also reflect their larger population bases. As provincial-level Weibo user data are not available for normalization, these findings should be interpreted as descriptive rather than indicative of differential emotional intensity across regions. The areas with intense emotional expression were not necessarily adjacent to the place where the event occurred, which was similar to the previous study by other Chinese researchers [38]. Based on a random sampling study, Weibo users were more likely to live in regions or provinces that were more economically developed [39]. Beyond the economic development explanation, other certain demographic or social factors (e.g., a higher concentration of MSM populations in some areas, such as Chongqing, Sichuan, and Shanghai) might also influence engagement levels, especially given the way mpox transmission was reported in the media [40]. It is suggested that the government should strengthen public opinion response and prevention in and control of areas with better economic conditions or a higher risk of disease importation. Additionally, public awareness campaigns and educational initiatives should be conducted in areas with lower levels of public discourse.

Based on the results of topic analysis and word frequency, users were most concerned about the transmission mode of mpox and showed a certain degree of resistance. Public preoccupation with transmission mode reflects underlying fear of contagion, a core component of risk perception. The media’s framing of mpox as disproportionately affecting MSM populations [41] may have amplified stigma-related concerns, contributing to the negative emotional tone observed in transmission-related discussions. The overwhelming predominance of negative emotions was consistent with patterns observed during other zoonotic disease outbreaks, including COVID-19 [20] and Ebola, suggesting this may be a characteristic public response to emerging infectious threats rather than a phenomenon unique to mpox. This negativity bias can be understood through risk perception theory, particularly the concepts of dread risk and uncertainty [42]. Dread risk refers to threats perceived as uncontrollable, catastrophic, and having fatal consequences, characteristics that apply to novel infectious diseases. Uncertainty about transmission routes, severity, and personal risk further amplifies anxiety, as individuals lack the information needed to accurately assess their vulnerability. It should be noted that some expressions of Internet slang, such as “退” (go away), and the reverse use of certain positive words, such as “谢谢” (thanks), were widely used. Owing to the influence of the diversification of online languages and the openness of social culture, traditional Chinese cultural terms that conveyed emotional nuances were increasingly replaced by neologisms or certain modal particles.

Our findings further confirmed that sentiment was closely related to the NC, NL, and NR, as many of the measures of post impact were constructed and calculated by them [14]. The strong correlation between comment volume and negative sentiment is consistent with the interpretation that comment counts may be useful as a practical heuristic for monitoring aggregate negative emotion in real time, although this does not imply a causal relationship. The finding with practical implications for digital surveillance, as comment volumes are immediately observable while sentiment analysis requires processing time. In terms of emotional guidance, topic richness can increase the NC, NL, and NR [43]. There are some other factors not included in our study that may affect the public’s emotions, such as role models and “rumors”. A role model is a spiritual carrier and embodiment that conveys positive emotions [38]. The existence of “rumors” can enable people to have more communication and discussion, which will have a negative psychological impact on the prevention and management of the epidemic [44].

Our findings have two key implications for risk communication during public health emergencies. First, the critical time window for intervention is extremely narrow. The peak in engagement and emotional intensity within the first four hours suggests that health authorities should issue proactive, myth-busting messages within 2–4 hours of an outbreak announcement to shape public perception before attention wanes and negative sentiment consolidates. Second, sentiment analysis can enhance real-time public health surveillance. The strong correlation between comment volume and negative emotional intensity suggests that social media engagement metrics could serve as early warning indicators of escalating public concern. Integrating automated sentiment analysis into public health dashboards would enable authorities to monitor emotional trends in real time, complementing traditional case-based surveillance and triggering timely communication responses when negative sentiment reaches predefined thresholds [45].

Several limitations of our study should be considered. Firstly, Sina Weibo users are not representative of the general Chinese population. The platform disproportionately attracts younger, urban, and tech-savvy individuals, and we were unable to obtain users’ age and sex information. Consequently, our findings primarily reflect the emotional responses of this specific demographic segment rather than the full spectrum of public opinion in China, limiting generalizability to older, rural, or less digitally connected populations. In addition, the provincial comment counts presented in Fig 3 were not normalized by provincial population or Weibo user base, as official provincial-level Weibo user statistics are not publicly available. The higher raw counts observed in developed regions such as Guangdong and Shanghai may partly reflect larger internet user populations rather than inherently greater emotional salience. Secondly, our analysis was restricted to direct comments on the top 40 most popular posts. While this approach captured the majority of public engagement (>95% of total interactions), it excluded secondary comments (replies to direct comments) and less popular posts. As noted in our original limitations, these excluded data may contain nuanced discourse, alternative perspectives, or emotional expressions that differ from those in mainstream discussions. Future studies should employ broader sampling strategies to capture a more complete picture of online discourse. Thirdly, although BosonNLP is widely used for Chinese social media sentiment analysis, dictionary-based methods have inherent limitations. The tool may not adequately capture sarcasm, irony, or culturally specific expressions such as internet slang and memes that convey emotional meaning indirectly. Moreover, while our stratified validation showed acceptable performance across content types, the lower agreement for internet slang suggested that dictionary-based methods may still misclassify some non-standard expressions. Future studies could consider deep learning approaches or integrating a custom slang lexicon to improve accuracy for such discourse features. Finally, the correlations observed between engagement metrics and EVs do not imply causation. While we interpret higher comment volumes as reflecting negative emotional arousal, it is equally plausible that negative emotions may drive more commenting. Additionally, unmeasured external factors, such as concurrent news events, government announcements, or rumors circulating on other platforms, may have influenced both engagement and emotional expression during the 48-hour observation period.

## 5. Conclusion

This study provides real-time insights into public emotional dynamics following the first imported mpox case in mainland China by analyzing 5,107 comments from Sina Weibo. We found that public sentiment was predominantly negative, peaked within the first four hours post-announcement, and was concentrated in economically developed regions. The strong correlation between comment volume and negative emotional intensity suggests that engagement metrics can serve as a proxy for aggregate sentiment. Methodologically, this study demonstrates the value of combining NLP with sentiment analysis to track real-time emotional responses during an emerging infectious disease event, which is a novel application in the Chinese social media context. Our findings underscore the need for health authorities to develop sentiment-aware communication protocols for emerging infectious diseases. Specifically, proactive, myth-busting messages should be issued within the first 2–4 hours of an outbreak announcement, and automated sentiment analysis should be integrated into public health dashboards to enable real-time monitoring of public concern and timely intervention.

## Supporting information

S1 Fig Flow diagram of data selection process. The diagram illustrates the step-by-step process from initial web crawling to the final dataset of 5,107 valid comments used for sentiment analysis. Inclusion and exclusion criteria, the justification for selecting the top 40 posts, and the data cleaning steps are shown.
(JPG)

S2 Fig Perplexity scores for LDA topic models with different numbers of topics (k). Perplexity scores across k values from 2 to 15. Lower perplexity indicates better predictive performance.
(JPG)

S1 Table Examples of words and their assigned emotional values from the BosonNLP dictionary. (DOCX)

S2 Table List of degree adverbs with assigned intensity weights (α). (DOCX)

S3 Table Coherence scores for LDA topic models with different numbers of topics (k). Coherence scores across k values from 2 to 15. Higher coherence scores indicate more semantically interpretable topics.
(DOCX)
