# Supplementary material for: Real-time emotional dynamics and public discourse on Sina Weibo following the first imported mpox case in mainland China: A sentiment analysis study
Source: PLoS One. 2026 Aug 7;21(8):e0354150. doi: 10.1371/journal.pone.0354150 (PMC13450724; doi:10.1371/journal.pone.0354150)
Supplement: Supplementary file 1 [file pone.0354150.s003.docx]

| **Positive emotions** | | | **Negative emotions** | | |
| --- | --- | --- | --- | --- | --- |
|  | Words | Values |  | Words | Values |
|  | Prosperous and thriving | 5.72 |  | Starving and freezing | -4.51 |
|  | Love forever | 5.72 |  | Fuck | -4.44 |
|  | Soft | 4.83 |  | Choke with resentment | -4.39 |
|  | Award | 4.55 |  | Help | -4.16 |
|  | Fabulous | 4.51 |  | Bad luck | -4.01 |
|  | Send love | 4.25 |  | Nightmare | -3.94 |
|  | Happy | 4.18 |  | Detestable | -3.90 |
|  | Discounts | 4.10 |  | Damn | -3.85 |
|  | Sweet | 2.64 |  | Shock | -2.48 |
|  | Esteem | 1.06 |  | Illness | -2.46 |
