# Supplementary material for: Real-time emotional dynamics and public discourse on Sina Weibo following the first imported mpox case in mainland China: A sentiment analysis study
Source: PLoS One. 2026 Aug 7;21(8):e0354150. doi: 10.1371/journal.pone.0354150 (PMC13450724; doi:10.1371/journal.pone.0354150)
Supplement: Supplementary file 2 [file pone.0354150.s004.docx]

| **Intensity level** | **Weight (α)** | **Degree adverbs** |
| --- | --- | --- |
| Superlative | 2.0 | Extremely, very much, most, most of all, absolutely, incomparably, utterly |
| Strongly positive | 1.8 | Very, especially, particularly, too, exceptionally, how |
| Moderately strong | 1.5 | Quite, really, truly, exceedingly, even more, further, more |
| Mild | 1.2 | Relatively, comparatively, rather, fairly |
| Weak | 0.5 | A little, somewhat, some |
| Slightly | 0.3 | Slightly |
