# Supplementary material for: Real-time emotional dynamics and public discourse on Sina Weibo following the first imported mpox case in mainland China: A sentiment analysis study
Source: PLoS One. 2026 Aug 7;21(8):e0354150. doi: 10.1371/journal.pone.0354150 (PMC13450724; doi:10.1371/journal.pone.0354150)
Supplement: Supplementary file 3 [file pone.0354150.s005.docx]

| **K values** | **Coherence_values** | **K values** | **Coherence_values** |
| --- | --- | --- | --- |
| 2 | -257.9544 | 9 | -221.1727 |
| 3 | -223.7222 | 10 | -226.8208 |
| 4 | -226.5451 | 11 | -228.8093 |
| 5 | -234.2089 | 12 | -216.1141 |
| 6 | -217.3777 | 13 | -219.4511 |
| 7 | -211.6173 | 14 | -215.6123 |
| 8 | -224.2331 | 15 | -216.1107 |
